# Knowing and Naming: Phage Annotation and Nomenclature for Phage Therapy

**DOI:** 10.1093/cid/ciad539

**Published:** 2023-11-02

**Authors:** Susanna R Grigson, Sarah K Giles, Robert A Edwards, Bhavya Papudeshi

**Affiliations:** Flinders Accelerator for Microbiome Exploration, College of Science and Engineering, Flinders University, Adelaide, Australia; Flinders Accelerator for Microbiome Exploration, College of Science and Engineering, Flinders University, Adelaide, Australia; Flinders Accelerator for Microbiome Exploration, College of Science and Engineering, Flinders University, Adelaide, Australia; Flinders Accelerator for Microbiome Exploration, College of Science and Engineering, Flinders University, Adelaide, Australia

**Keywords:** Bacteriophages, antibiotic resistance, plaque morphology, genomic features, protein-coding sequences, Taxonomy

## Abstract

Bacteriophages, or phages, are viruses that infect bacteria shaping microbial communities and ecosystems. They have gained attention as potential agents against antibiotic resistance. In phage therapy, lytic phages are preferred for their bacteria killing ability, while temperate phages, which can transfer antibiotic resistance or toxin genes, are avoided. Selection relies on plaque morphology and genome sequencing. This review outlines annotating genomes, identifying critical genomic features, and assigning functional labels to protein-coding sequences. These annotations prevent the transfer of unwanted genes, such as antimicrobial resistance or toxin genes, during phage therapy. Additionally, it covers International Committee on Taxonomy of Viruses (ICTV)-an established phage nomenclature system for simplified classification and communication. Accurate phage genome annotation and nomenclature provide insights into phage–host interactions, replication strategies, and evolution, accelerating our understanding of the diversity and evolution of phages and facilitating the development of phage-based therapies.

Bacteriophages (hereafter, phages) are incredibly prevalent in the environment, with an estimated 10^31^ phages present [1] across every environment where bacteria exist, including soil, water, and the human body. Phages were first isolated to target pathogens and widely used to treat bacterial infections in the early twentieth century. However, in the post-war era, antibiotics’ broad-spectrum activity displaced phages, and interest in them waned. In the early twenty-first century, the rampant spread of antibiotic-resistant microbes that abrogate the effects of antibiotics has led to renewed interest in using phages as therapeutic agents [2, 3].

Phages typically sustain two lifestyles, temperate and virulent, determined by their genetics and interactions with bacterial hosts. Temperate phages integrate within the bacterial genome as prophages, establishing long-term lysogenic relationships. However, integrated phages (prophages) can transition to lytic replication under certain conditions or triggers [4]. Temperate phages are unsuitable for phage therapy due to their potential to transfer antibiotic resistance, virulence genes, or pathogenicity islands to other bacteria [5–7]. Additionally, lysogeny often leads to superinfection exclusion, where the prophage protects the host from infection by other closely related phages [8, 9]. Conversely, virulent phages use lytic replication that induces the host cell’s death post-infection. Therefore, obligate lytic phages are preferred for phage therapy, as they pose reduced susceptibility to confer phage resistance.

Isolated phages are first screened based on plaque morphology, aiming to select lytic phages that display distinct, clear plaques. Subsequently, these phages are sequenced to decipher their genetic makeup and identify their genes. However, 65% of viral genes are functionally unassigned [10]. To address this, continued efforts are being made to develop novel tools to leverage phage-specific characteristics, facilitating the identification of protein-coding regions and advancing the prediction of their biological functions. These annotations are pivotal in determining phage interactions, replication, evolutionary dynamics, and host range. Accurate genome annotation also ensures that phages do not transfer unwanted genes, such as antimicrobial resistance genes and toxins, to new environments. Simultaneously, this process allows for selecting genomes with depolymerases that provide antimicrobial and antibiofilm activities [11–13]; anti–clustered, regularly interspaced, short palindromic repeats (CRISPRs); and antitoxin genes that help phages overcome bacterial immune systems [14]. To comprehensively assess phages’ therapeutic potential and diversity, we classify them into the suitable taxonomy to place the phage in an evolutionary context.

In this review, we outline the current procedures for annotating and naming phage genomes (Figure 1). We provide an overview of lytic phage detection, genome assembly, and structural and functional annotations (Table 1). We highlight the critical proteins for determining a phage’s efficacy for phage therapy and describe the established phage nomenclature system and the procedures for assigning an appropriate name to a phage that adheres to standardized naming conventions. This resource allows for precise phage identification, categorization, and comprehension, bolstering the advancement of phage-based therapies and biotechnologies.

**Figure 1.**
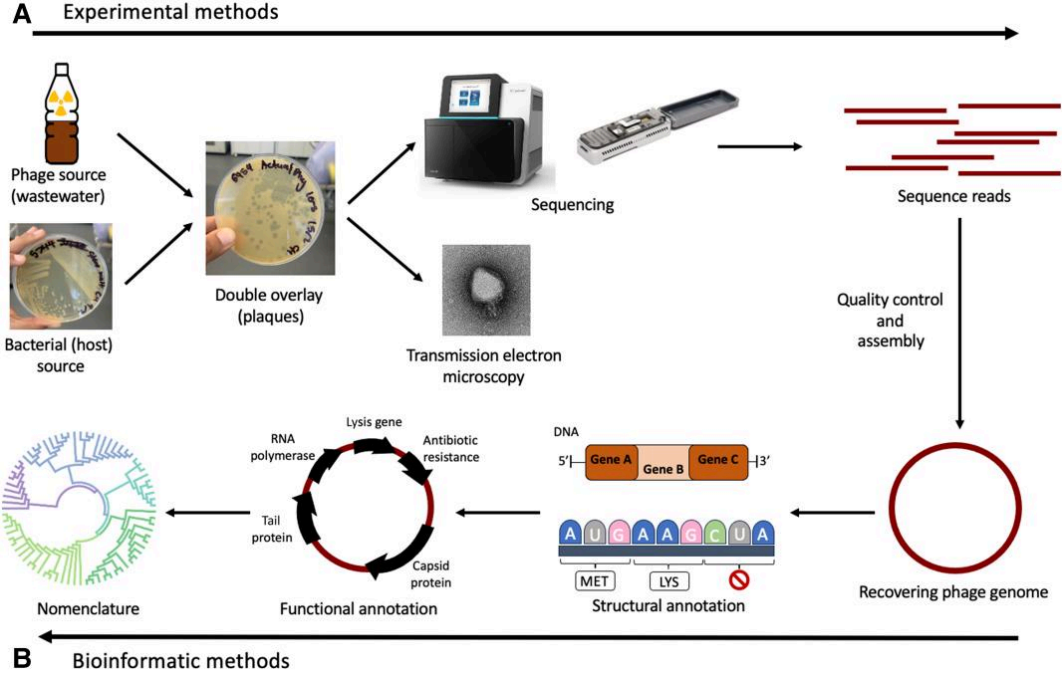
Overview of the steps in phage isolation and characterization. *A*, Experimental methods: the double overlay method facilitates plaque formation, helping isolate and select lytic phages from an environmental source. Transmission electron microscopy helps visualize the isolated phages to determine the broad taxonomic grouping. Concurrently, the process involves extracting the isolated phage’s DNA and subjecting it to sequencing. *B*, Bioinformatics methods: assembly of sequence reads allows for the recovery of complete genomes, accurate annotation, and phylogenetic classification. Abbreviations: LYS, lysine; MET, methionine.

**Table 1.** Summary of the Bioinformatics Tools Used for Phage Assembly and Annotation Described in This Review

Category	Name	Brief Overview	Github	Reference
Quality control	Prinseq++	Quality control steps of sequenced reads	https://github.com/Adrian-Cantu/PRINSEQ-plus-plus	[15]
	Filtlong	Quality control steps of Nanopore sequenced reads	https://github.com/rrwick/Filtlong	[16]
Assembly	MetaViralSPAdes	Assembling viral sequences	https://github.com/ablab/spades	[17]
	ViralFlye	Assembling viral sequences	https://github.com/Dmitry-Antipov/viralFlye	[18]
Phage quality assessment	CheckV	Phage genome completeness	https://bitbucket.org/berkeleylab/checkv/src/master/	[19]
	viralCompletete	Phage genome completeness	https://github.com/ablab/viralComplete	…
	MMSeqs2	Map the reads to the phage genome, to determine even genome coverage	https://github.com/soedinglab/MMseqs2	[20]
Phages from metagenomes	Phables	Identify genome termini signals from assembly graphs	https://github.com/Vini2/phables	[21]
Phages from bacterial genomes	PhiSpy	Prophage identification from bacterial genomes	https://github.com/linsalrob/PhiSpy	[22]
Phage structural annotation	PHANOTATE	Gene identification in phage genomes	https://github.com/deprekate/PHANOTATE	[23]
	Prodigal	Gene identification in prokaryotic genomes	https://github.com/hyattpd/Prodigal	[24]
	PRFect	Predict ribosomal frameshifting	https://github.com/deprekate/prfect	[25]
	tRNAscanSE	Detection and functional classification of transfer RNA genes	http://lowelab.ucsc.edu/tRNAscan-SE/	[26]
	ARAGORN	Detection of mRNA and tRNA genes	http://www.ansikte.se/ARAGORN/	[27]
	Mgcod	Accurate annotation considering stop codon reassignment	https://github.com/gatech-genemark/Mgcod	[28]
Phage orthologous cluster gene database	PHROGs	Prokaryotic virus proteins database	https://phrogs.lmge.uca.fr/	[29]
	Prokaryotic virus orthologous groups	Prokaryotic virus orthologous groups database	ftp://ftp.ncbi.nlm.nih.gov/pub/kristensen/pVOGs/home.html	[30]
Phage functional annotation	mmseqs	Map the proteins to a phage protein database	https://github.com/soedinglab/MMseqs2	[20]
	HMMER	Probabilistic models to predict the phage protein annotation	https://github.com/EddyRivasLab/hmmer	[31]
	PVP-SVM	Prediction of phage virion proteins	www.thegleelab.org/PVP-SVM/PVP-SVM.html	[32]
	DeePVP	Classification of phage structural proteins	https://github.com/fangzcbio/DeePVP	[33]
	PVPred-SCM	Prediction of phage virion proteins	https://github.com/Shoombuatong/PVPred-SCM	[34]
	VirionFinder	Prediction of phage virion proteins	https://github.com/zhenchengfang/VirionFinder	[35]
	PhANNs	Classification of phage structural proteins	https://github.com/Adrian-Cantu/PhANNs	[36]
	AlphaFold	Protein structure prediction	https://github.com/deepmind/alphafold	[37]
	ColabFold	Protein structure prediction	https://github.com/sokrypton/ColabFold	[38]
	FoldSeek	Protein structure search	https://github.com/steineggerlab/foldseek	[39]
	AMRFinderPlus	Antimicrobial resistance gene search	https://github.com/ncbi/amr	[40]
	CARD	Antimicrobial resistance gene search	https://card.mcmaster.ca/	[41]
	VFDB	Virulence factor database search	http://www.mgc.ac.cn/VFs/	[42]
	BACPHLIP	Bacteriophage lifestyle prediction	https://github.com/adamhockenberry/bacphlip	[43]
	PHACTS	Bacteriophage lifestyle prediction	https://github.com/deprekate/PHACTS	[44]
	RaFAH	Host prediction	https://sourceforge.net/projects/rafah/	[45]
	Pharokka	Phage annotation pipeline	https://github.com/gbouras13/pharokka	[46]
	MultiPhaTE2	Phage annotation and comparative analyses	https://github.com/carolzhou/multiPhATE2	[47]

## FINDING LYTIC PHAGES

**Figure 2.**
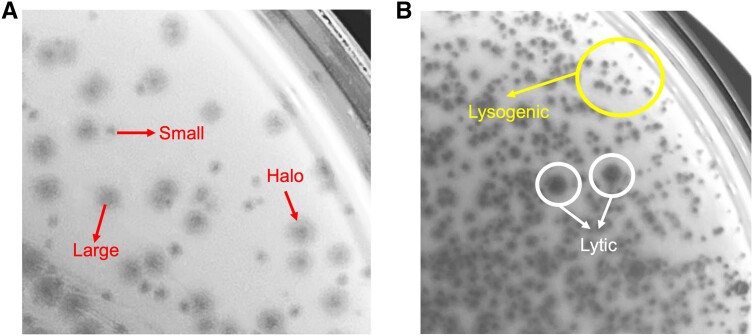
Distinct plaque morphologies using the double overlay method. *A*, Plaques from an environmental sample display a halo and are of variable sizes, large (>3 mm in diameter) or small (1 mm in diameter), denoting different phage species. *B*, Co-occurrence of lytic and lysogenic phage phenotypes in subsequent phage isolation. Lytic phages form large, clear plaques, and lysogenic phages form cloudy plaques.

Typically, we collect environmental samples rich in bacteria, such as soil, water, or sewage, to isolate phages. We start by enriching and filtering to concentrate phages within a sample [48]. We enrich the phages by coculturing bacterial and environmental samples, which amplify environmental phages, resulting in higher plaque counts. We use the commonly used double overlay method [49] to visualize, isolate, and purify phages by plaque formation, permitting the identification of diverse morphologies. Subsequently, we pick single plaques and culture them to separate distinct phage species from environmental samples. A second round of plaquing allows us to distinguish between large (>3 mm diameter) and small (1 mm in diameter) plaque sizes (Figure 2*A*). Other morphological features include halos around the plaque, which indicate endonuclease activity (Figure 2*A*). Interestingly, infection by a lytic phage can induce prophages, leading to the co-occurrence of lytic and lysogenic phage phenotypes in a single, double overlay plate (Figure 2*B*). To identify lytic phenotypes, plate-based methods such as cross-streaking [50]and detecting spontaneous phage release using spot and immunity assay techniques [51] can assist in identifying temperate phenotypes.

After successive rounds of purification, we enumerate phages by serial dilution and titration. We visualize the phage by transmission electron microscopy and broad taxonomic groupings. Subsequently, we grow phages in large quantities to extract DNA for sequencing. Although short-read sequencing technologies are more commonly used to assemble phage genomes, there has been a growing interest in using long-read sequencing. A recent study showed that long-read assemblies generated higher-quality, complete genome assemblies; however, they required polishing with short-read sequencing to correct frameshift errors [52]. We aim for 25 times to 100 times coverage during sequencing because higher coverage often causes assembly failures due to too many single-nucleotide polymorphisms in the data [53]. Post-sequencing, the first step is to trim the DNA sequences through quality control [15, 16] before assembling them to recover complete phage genomes [17, 18]. Then, we evaluate the phage genomes’ quality, as low-quality phage genomes prevent accurate annotation. To evaluate genome quality, reads are mapped back to the assembled genome using MMseqs2 [20], we consider the distance between paired ends, the mapping orientation of reads, and an even read coverage across the genome [53, 54]. We further test for genome completeness using tools such as CheckV [19]. Although we check for direct terminal repeats (DTRs) to indicate terminal redundancy [55, 56], the assembly process often obfuscates the genome termini signals. Furthermore, long-read assemblies, in combination with short-read assemblies, can resolve phage DTRs [57]. Alternatively, our Phables algorithm explores bubbles in the assembly graph and highlights and corrects terminal redundancy [21].

After assembly, we use computational methods that include genome information to anticipate whether a phage has a virulent or temperate lifestyle. These methods encompass sequence similarity–based random forest classifiers [44], the presence of specific protein domains [8], and a hybrid approach that uses both types of information [43].

## KNOWING PHAGE GENETIC POTENTIAL

Having successfully isolated, sequenced, and assembled a phage genome, we start by characterizing the structural features to unpack their functional properties (Figure 1).

### Structural Annotation

The first step involves identifying open reading frames (ORFs), which encode proteins and other elements such as tRNAs, noncoding RNAs, promoters, and transposons. Gene-calling algorithms use codon usage and Guanine-Cytosine content biases, start codons, stop codons, Shine–Delgarno sequences, and short nucleotide sequences that bind to ribosomes during protein translation to identify open-reading frames [23, 24]. Unique characteristics of phage genomes, such as higher coding capacity, shorter intergenic regions, and more overlaps between coding domain sequences than bacterial genomes, require phage-specific tools [22, 58]. We built PHANOTATE to exploit these idiosyncrasies and improve phage gene identification [23]. Additionally, phages use several tricks to expand their genetic arsenal, such as programmed ribosomal frameshifting that allows phages to make two versions of a protein by switching to an alternate coding strand [25].

Like ORFs, identifying transfer RNA (tRNA) genes combines sequence and structural features. Tools such as tRNAscanSE [26] and ARAGORN [27] identify RNA-encoding genes before ORF calling begins. Some phages reassign stop codons [59–61] to encode amino acids, a strategy to modulate translation and prevent premature host cell lysis. Further, phage genomes can feature heavily modified and alternative nucleotides, aiding phages in extracting more energy from their bacterial hosts and for protection against defense systems [62]. Long-read sequencing technologies offer another advantage, with single-molecule, real-time sequencing (PacBio SMRT) and Oxford Nanopore sequencing being able to detect DNA modifications directly [63].

### Functional Annotation

After identifying the protein- and RNA-encoding regions, the next step is to assign biological functions to each gene. Despite the fact that phages have smaller genomes than bacteria, it remains challenging to ascribe functions, as 65% of viral protein sequences lack known biological function [10].

Phage genes typically encode proteins in one of several categories of functions as follows: First, structural and packaging proteins include capsid, baseplate, and tail fiber proteins. These proteins form the outer capsid of the free phage. Second, phage integration, excision, and maintenance of the integrated state, including recombination and DNA binding proteins. Third, DNA replication proteins, including DNA polymerases and single-stranded binding proteins. Fourth, accessory metabolic genes that often provide temporary metabolic boosts to the cell to increase energy production while the phage is replicating. Finally, morons and genes of unknown function [64].

Three popular databases target those clusters. PHROGs (prokaryotic virus remote homologous groups) [29], VOGs (viral orthologous groups), and pVOGs (prokaryotic virus orthologous groups) [30] build clusters of orthologous genes with shared functions. Orthologous genes have arisen by speciation (vertical transmission), and these databases attempt to disambiguate orthologs from proteins that evolved by duplication (paralogs) and thus may have similar but different functions. The PHROGs database is unique because it assigns each orthologous group to 1 of 9 categories, allowing two levels of annotation. While phage genes are assigned an orthologous group, many have unknown functions; for example, 87% (33 747 out of 38 880 orthologous groups) of PHROGs lack a function. Most phage annotation pipelines use homology searches (eg, with MMSeqs2 [20], HMMER [31], or HHpred [65]) to search each coding domain sequences with these databases.

Among the protein categories, structural proteins receive more annotations than the others due to their prominent role in the phage life cycle and direct involvement in host interactions and the external environment. Efforts to distinguish these structural proteins from those that serve alternative functions have involved various approaches, including using classifiers built with support vector machines [32–35]. We further expanded on these methodologies by training an artificial neural network to discern 10 distinct classes of proteins, including primary and minor capsid proteins, based on their sequence composition [36]. Recent advancements in artificial intelligence have introduced large language models that take PHROG cluster sequences as input and derive protein functional properties from the embedded amino acid sequences to improve protein annotations [66]. Another approach to improve annotations has been through the prediction of the 3-dimensional protein structures and structure-based predictions [37, 38]. For instance, Say et al used Colabfold [38] to predict structures and used Foldseek [39] to search a database [67]. These emerging strategies improve functional annotations and deepen their biological and practical context.

### Examining Phage Suitability

In the context of phage therapy, specific genes are pivotal in determining if the phage is a potential candidate for phage therapy. For instance, “integrase” annotated genes are not preferred as they are markers for temperate lifestyles. While specific gene functions may confer phage therapy advantages, this includes phage-encoded depolymerase genes, which degrade specific components of a bacterial surface, including extracellular polysaccharides and biofilm matrices [68]. Depolymerase activity can present as a halo around the plaques (Figure 2*A*), and this gene activity makes the bacteria more susceptible to host infection, improving access to bacterial surfaces and reducing antibiotic dependence. Annotation pipelines may label these genes as “depolymerase,” “host-interaction protein,” “capsule degrading enzyme,” “biofilm matrix-degrading enzyme,” or similar [69–71].

Additionally, tail spike and tail fiber proteins include phage receptor binding proteins (RBPs), which are crucial for host interactions and indicative of host specificity. Often, the annotations list these proteins as “receptor-binding protein,” “receptor-recognizing protein,” or similar variations [72]. Tools such as PhageRBP have developed models based on detected RBP sequences and protein domains to see these proteins in the phage isolated [73]. Experimentally, techniques such as the efficiency of plating, which quantifies plaque-forming efficiency and burst size, representing the number of progeny virions released per infected host cell, also characterize phage–host interactions [74].

Other relevant genes include anti-host systems that phages develop to counter bacterial defense mechanisms against phage infections. Phages adapt by mutating RBPs at a high frequency in an order that is mediated by the activity of diversity-generating retroelements [75, 76] to recognize the bacterial host. To bypass the restriction-modification systems in the bacterial hosts, phages alter their restriction sites using nucleotide modifications or reorientation [77]. In response to bacterial immunity mechanism, abortive infection mechanisms, which involve toxin–antitoxin action, phages inhibit the antitoxin degradation or produce antitoxin analogs to defend themselves. Finally, to evade clustered, regularly interspaced, short palindromic repeats (CRISPR/Cas), phages modify palindrome repeats or use anti-CRISPRs (*Acr*) proteins that hinder the system’s activity. Programs such as minCED identify CRISPR spaces in the phage genome [78].

Interestingly, phage resistance can develop quickly in vitro, but the results are variable in vivo [77]. Unfortunately, while we can identify and examine the phage for known anti-host defense systems, several mechanisms still need to be identified. Mechanisms that underlie phage resistance are seldom studied; instead, there is more focus on clinical outcomes, in particular, on phage cocktails or combination therapy (antibiotics with phages) to overcome phage resistance.

Moreover, the potential for phages to routinely transfer antibiotic resistance genes in the environment requires further clarification. Studies exhibit varying outcomes; some indicate increased antibiotic resistance gene transfer [13, 79], while others present opposing results [80]. Similarly, many phages are known to carry toxins, so we use virulence factor databases to identify gene products that might be involved in pathogenesis, including toxins [81, 42]. Therefore, aligning phage genes against boutique databases with highly accurate, manually curated data such as the National Center for Biotechnology Information (NCBI) AMRFinderPlus [40] and comprehensive antibiotic resistance database(CARD) [41] virulence factor databases [42] is necessary.

### Genome Annotation and Comparison Workflows

Bioinformatics workflows include the multistep process described above to annotate phages. Pharokka [46] layers the annotations, providing more weight to boutique annotations than similarity searches against large databases. Pharokka generates the files required to submit a new genome sequence to GenBank but also creates visualizations and provides output formats suitable for comparing genomes. In contrast, we designed MultiPhaTE2 to handle multiple phage genomes and perform comparative analyses [47] in order to understand phage diversity and dynamics.

## NAMING A PHAGE

### Phage Nomenclature

Most biological organisms are named using a binomial nomenclature and a standard taxonomic hierarchy. The International Committee on the Taxonomy of Viruses (ICTV) is the authoritative committee for classifying viruses. It delineates that classification into 15 taxonomic ranks between realm and species [82]. The ICTV ratifies viral nomenclature, and the Bacterial and Archaeal Viruses Subcommittee (BAVS) is responsible for phage nomenclature. The primary requirement for ratifying a new phage is depositing a complete genome sequence in 1 of the 3 International Nucleotide Sequence Database Collaboration (INSDC) member databases [83]. By 2022, the ICTV had ratified 6 realms, 10 kingdoms, 17 phyla, 2 subphyla, 40 classes, 72 orders, 8 suborders, 264 families, 182 subfamilies, 2818 genera, 84 subgenera, and 11 273 species [84].

## Naming Guidelines

The first step in naming a phage is to invent a novel name for the isolate. There are several guidelines for prescribing phages (eg, the SEA-PHAGES program has a set of rules, https://phagesdb.org/namerules/, and members of the ICTV BAVS published an informal guide to choosing a name [85]). The guidelines are similar: do not use an existing phage name or one similar to a current name, keep the name short (about 5 to 15 characters), and do not start with a number.

The next step is to determine its novelty compared with known phage sequences. We usually compare the phage’s genome sequence to the phage genomes that are in existing databases. The Millard Lab maintains and regularly updates a list of complete phage genomes (https://millardlab.org/). The ICTV guidelines suggest that novel species are more than 5% different from existing species at the nucleotide level. In comparison, novel genera are more than 50% distinct from existing genera at the nucleotide level. Unique characteristics distinguish unknown taxonomic groups, including genome length, number of coding sequences, and phylogenetic clustering of marker genes such as portal protein, large terminase, and significant capsid genes. High-level classifications (eg, subfamily or family) still require an electron micrograph to classify a phage into an appropriate group based on its morphotypes.

When identifying a new phage, one should determine the highest possible taxonomic classification by sequence similarity and explore additional characteristics to help assign a proper name. For example, we recently described 3 new phages that infect *Bacteroides cellulosilyticus*. The phages’ genome lengths and podovirus-like morphologies suggested they belonged to the *Crassvirales* order, and their sequence similarity to other crAss-like phages further supported this [52]. Phylogenetic clustering and nucleotide similarity searches confirmed that 2 genomes belonged to known genera but were distinct enough to be considered new species. The third phage was unlike any known crAss-like phages, so we proposed it as a new genus using the templates on the ICTV website (https://ictv.global/taxonomy/templates).

A complete list of guidelines for naming phages is available on the ICTV website (https://ictv.global/about/code). If the isolated phage does not fit the current known taxonomic groups genomically or morphologically, the ICTV will help define a new category.

## SHARING PHAGES

Making the bacteriophage genomes available through 1 of the 3 public INSDC repositories (National Center for Biotechnology Information [NCBI], DNA Data Bank Japan [DDBJ], or European Nucleotide Archive [ENA]) is good practice. It is required when publishing the work in any peer-reviewed journal. Each of the 3 databases has an online portal where complete, annotated genome sequences can be submitted.

Depositing the phage isolates to specialized repositories such as the American Type Culture Collection; National Collection of Industrial, Food, and Marine Bacteria; and Phage Australia facilitates access to these phages. Alternatively, local collections such as Phage Australia provide a platform for making phage information and their characteristics publicly accessible.

## CONCLUSIONS

Phage annotation and nomenclature are essential for precisely identifying and characterizing phages. They also facilitate communication and sharing of research data. Proper nomenclature and annotation ensure consistency and clarity in naming and describing phages, which is crucial for understanding their biological properties, host range, and genetic makeup. It also allows for comparing and integrating data from different studies, thus facilitating progress in phage biology and biotechnology. Careful phage annotation is a prerequisite for phage therapy.

## Notes

***Author Contributions.*** S. R. G., S. K. G., R. A. E., and B. P. wrote the article.

***Financial support.*** This work was supported by awards from the National Institutes of Health (NIH) National Institute of Diabetes and Digestive and Kidney Diseases (NIDDK) (RC2DK116713), the Australian Research Council (DP220102915), and the Gordon and Betty Moore Foundation (9871; Perpetual Viral Origins). A Playford Trust PhD Scholarship supported S. R. G. Grant funding from the Royal Society of South Australia (grant RPF21/448) supported S. K. G.

***Supplement sponsorship.*** This article appears as part of the supplement “Personalized Phage Therapy: Basic Principles of Monitoring and Treatment,” sponsored by Adaptive Phage Therapeutics, Mayo Foundation for Medical Education and Research, BiomX, Technophage Investigação E Desenvolvimento Em Biotecnologia S A, and by ESCMID Study Group for Non-traditional Antibacterial Therapy (ESGNTA).
